# Improved Protein Removal Performance of PES Hollow-Fiber Ultrafiltration Membrane with Sponge-like Structure

**DOI:** 10.3390/polym16091194

**Published:** 2024-04-25

**Authors:** Huyang Zhao, Ting He, Shuang Yao, Long Tao, Xinhai Zhang, Zhaohui Wang, Zhaoliang Cui, Rizhi Chen

**Affiliations:** 1 State Key Laboratory of Materials-Oriented Chemical Engineering, College of Chemical Engineering, Nanjing Tech University, Nanjing 210009, China; 202161104209@njtech.edu.cn (H.Z.); tinglei@njtech.edu.cn (T.H.); 17680395516@163.com (L.T.); nights0921@gmail.com (X.Z.); rizhichen@njtech.edu.cn (R.C.); 2 National Engineering Research Center for Special Separation Membrane, Nanjing Tech University, Nanjing 210009, China; 3 Jiangsu National Synergetic Innovation Center for Advanced Materials (SICAM), Nanjing Tech University, Nanjing 210009, China

**Keywords:** hollow-fiber membrane, polyethersulfone, non-solvent-induced phase separation, protein separation

## Abstract

The research used polyethersulfone (PES) as a membrane material, polyvinylpyrrolidone (PVP) k30 and polyethylene glycol 400 (PEG 400) as water-soluble additives, and dimethylacetamide (DMAc) as a solvent to prepare hollow-fiber ultrafiltration membranes through a nonsolvent-induced phase separation (NIPS) process. The hydrophilic nature of PVP-k30 and PEG caused them to accumulate on the membrane surface during phase separation. The morphology, chemical composition, surface charge, and pore size of the PES membranes were evaluated by SEM, FTIR, zeta potential, and dextran filtration experiments. The paper also investigated how different spinning solution compositions affected membrane morphology and performance. The separation efficiency of membranes with four different morphologies was tested in single-protein and double-protein mixed solutions. The protein separation effectiveness of the membrane was studied through molecular weight cutoff, zeta potential, and static protein adsorption tests. In addition, the operating pressure and pH value were adjusted to improve ultrafiltration process conditions. The PES membrane with an intact sponge-like structure showed the highest separation factor of 11, making it a prime candidate membrane for the separation of bovine serum albumin (BSA) and lysozyme (LYS). The membrane had a minimal static protein adsorption capacity of 48 mg/cm^2^ and had excellent anti-fouling properties. When pH = 4, the BSA retention rate was 93% and the LYS retention rate was 23%. Furthermore, it exhibited excellent stability over a pH range of 1–13, confirming its suitability for protein separation applications.

## 1. Introduction

**Figure 1:**
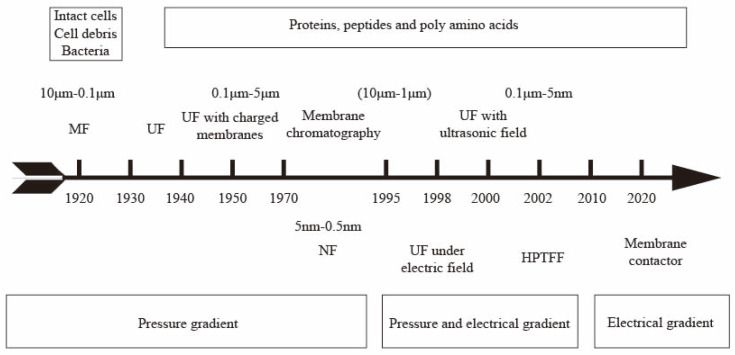
Milestones in the development of membrane technologies for protein separation/purification.

Protein separation and purification technologies play a key role in fields such as the pharmaceutical industry, food processing, and biotechnology [1,2]. Traditional protein separation and purification techniques, like extraction, precipitation, and centrifugation, have been extensively used. However, they still have many limitations, including protein denaturation and hydrolysis during extraction and precipitation and choosing an appropriate centrifugation resolution [3,4,5]. The difference in protein size is the basis for protein separation. Typically, biomolecules in the biopharmaceutical field, such as proteins, enzymes, and nucleic acids, have dimensions of less than 10 nm when their molecular weight is less than 1000 kDa. Viruses have a size of about 30 nm and a molecular weight of about 10,000 kDa [6]. It has been reported that the pore size of ultrafiltration membranes can be controlled to 2–50 nm depending on the manufacturing process, making them suitable candidates for the separation or immobilization of enzymes and proteins [7]. Protein purification protocols typically involve a combination of techniques, including precipitation, centrifugation, ion exchange, membrane separation, and gel chromatography. Ultrafiltration membrane filtration techniques are often studied with a focus on the protein molecular size property [8]. However, when designing protocols for protein purification, it is important to consider the use of different protein properties while minimizing the number of purification steps to improve efficiency [9]. Specifically, when using ultrafiltration membrane filtration, it may be beneficial to explore whether purification can be optimized by adjusting the pH of the solution based on the difference in isoelectric points of different proteins. This approach has the potential to lead to new breakthroughs in the field of protein purification. The application progress of membrane separation techniques such as microfiltration, ultrafiltration, and membrane chromatography in protein separation [9,10,11,12,13,14,15] is shown in Figure 1. Ghosh et al. studied the purification of LYS from chicken egg white using hollow-fiber PES UF membrane (30 kDa MWCO) [16]. The UF of fermented cheese whey broth was also studied using a lab-scale cross-flow membrane system with PES membranes (5, 20 kDa MWCO) [17]. Separation of β-lactoglobulin (LG) from whey protein was achieved by fractionation using two-stage UF with PES membrane (30 and 10 kDa MWCO) in a stirred rotating disk module followed by ion-exchange membrane chromatography [18]. Compared to traditional separation methods, membrane separation can operate at lower temperatures and pressures, preserve protein activity, and have additional benefits such as high separation efficiency, a small footprint, low chemical consumption, and easy scaling [19]. However, the hydrophobicity of most membrane materials can lead to membrane fouling, significantly affecting membrane separation performance [20]. Optimizing the membrane production process and operating conditions is crucial to achieving stable membrane flux and high selectivity for industrial-scale production [12].

Recently, attention has been paid to the preparation of ultrafiltration membranes, and the most popular fabrication method is non-solvent-induced phase separation (NIPS) [21]. Compared with complex membrane preparation methods, this method has lower requirements on conditions and can be produced on a large scale. By tuning the thermodynamic and kinetic behavior of the spinning solution during the NIPS process, target membranes with tailored structures and properties can be obtained in one step. Polyethersulfone (PES) is frequently utilized for producing ultrafiltration membranes due to its cost-effectiveness and excellent resistance to acids, alkalis, and chlorine among various polymers [22]. Due to its impressive biocompatibility and limited vulnerability to side effects such as coagulation and hemolysis, it is recognized as one of the superior membrane substances for the separation of biomass materials [23,24,25,26]. However, it is challenging to produce ultrafiltration membranes with low protein adsorption and dense, small-pore-structure-utilizing pure PES spinning solution in the NIPS process [27]. Adding hydrophilic additives to the spinning solution can effectively improve the NIPS process while changing the surface properties of the membrane, which is a strategy to build a thin and dense surface layer with high hydrophilicity [28,29,30]. Wang and colleagues found that in PES membranes containing PVP, water flux and hydrophilicity were greatly enhanced, while BSA adsorption was reduced [31]. Furthermore, the addition of PVP-k30 increased the viscosity of the spinning solution and improved the mechanical properties of the membrane. Consequently, the PES/PVP/PEG composite system is considered a promising competitor for the production of ultrafiltration membranes for protein separation. Al Malek and colleagues prepared PES membranes by dissolving 15% and 20% PES and 0–25% PVP in NMP. The study found that after the introduction of PVP into the spinning solution, the membrane permeability increased significantly. When the concentration of PVP and PES was 15 wt%, the maximum water permeability reached 376.8 L·m^−2^·h^−1^ [32].

In this study, a high-performance hollow-fiber membrane was prepared using PES/PVP/PEG blends as spinning solutions. The structure and properties of the membrane were fully characterized, including its morphology, chemical composition, and surface charge properties. The aim was to study the effect of additives on membrane morphology and performance. Membrane separation performance was extensively evaluated in a binary system containing BSA and LYS. This membrane manufacturing technology is expected to introduce new methods for producing flexible, high-performance hollow-fiber membranes for protein separation, thereby facilitating the scale-up of production.

## 2. Experimental Materials and Methods

### 2.1. Materials

Polyethersulfone (PES, 3000 MP; Solvay (Shanghai) Co., Ltd., Shanghai, China) was dried in a vacuum drying oven at 60 °C before use. The additive polyvinylpyrrolidone (PVP-k30) was purchased from Sinopharm Chemical Reagent Co., Ltd., Shanghai, China, and polyethylene glycol (PEG, Mw = 400) was purchased from Shanghai Aladdin Biochemical Technology Co., Ltd., Shanghai, China. N,N-dimethylacetamide (DMAc, analytical grade; Shanghai Aladdin Biochemical Technology Co., Ltd.) was used as a solvent to dissolve the polymer. Bovine serum albumin (BSA, analytical grade; Shanghai Huixing Biochemical Reagent Co., Ltd., Shanghai, China), lysozyme (LYS, analytical grade; Shanghai McLean Biochemical Technology Co., Ltd., Shanghai, China), sodium chloride (NaCl, analytical grade; Shanghai Aladdin Biochemical Technology Co., Ltd.), potassium chloride (KCl, analytical grade; Shanghai Lingfeng Chemical Reagent Co., Ltd., Shanghai, China), potassium dihydrogen phosphate (KH_2_PO_4_, analytical grade; Shanghai Lingfeng Chemical Reagent Co., Ltd.), and disodium hydrogen phosphate dodecahydrate (analytically pure; Xilong Chemical Co., Ltd., Yulin, China) were used to test the membrane separation performance. Membrane stability was tested using hydrochloric acid (HCl, ACS reagent, 37%) and NaOH. Neutral organic solute dextran (Shanghai McLean Biochemical Technology Co., Ltd.) with different molecular weights of 10,000, 70,000, 100,000, 150,000, and 500,000 Da was used for membrane molecular weight cutoff characterization. All chemicals were used without further purification unless otherwise stated.

### 2.2. PES Hollow-Fiber Membrane Preparation

**Figure 2:**
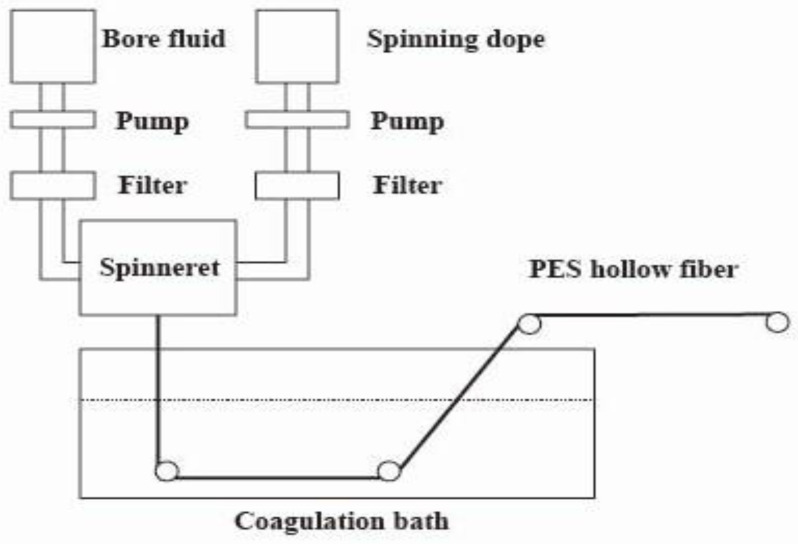
PES hollow-fiber spinning apparatus.

Hollow-fiber membranes were prepared using the NIPS process. Table 1 lists the composition of the spinning solution. Adding PEG and PVP-k30 to the spinning solution can enhance the hydrophilicity of the membrane, thereby reducing the protein adsorption capacity. In order to prepare the spinning solution, a predetermined amount of dried PES, PVP-k30, and PEG was poured into DMAc and stirred at 60 °C for about 10 h until completely dissolved. The resulting solution was clear and transparent. Then it was poured into the feed tank of the spinning machine. In addition, the exhaust valve above the feed tank was opened. The bore liquid tank temperature was set to 25 °C, and the spinning solution tank temperature was set to 60 °C. After the spinning solution was poured into the solution tank, the item was allowed to remain undisturbed for a minimum of 24 h. Figure 2 shows the preparation process of hollow-fiber membranes. Table 2 shows the corresponding process parameters and operating conditions.

After degassing the spinning solution, the spinning process commenced. The coagulation bath was filled with pure water, and the bore liquid was inspected to verify that the spinneret was unobstructed. The spinning solution tank was pressurized using a nitrogen cylinder regulated to approximately 0.1 MPa through the cylinder pressure-reducing valve and regulator. It is crucial to note that the pressure in the spinning solution tank not only impacts the spinning speed but also influences the inner and outer diameters of the hollow-fiber membrane.

The bore liquid was supplied from a bore liquid tank pressurized by a nitrogen cylinder. Since the bore liquid flow rate is usually very small, a slight change in pressure will affect the spinning stability; therefore, in this experiment, a bore liquid pump (peristaltic pump model: X-900-0.6cc; Guangzhou Moni Pump Mechanical and Electrical Equipment Co., Ltd., Guangzhou, China) and a spinning solution pump (peristaltic pump model: X-900-0.6cc; Guangzhou Moni Pump Mechanical and Electrical Equipment Co., Ltd.) were used to control the bore liquid and spinning solution flow rates, respectively. The spinning solution in the tank was extruded into the spinneret through the pump. The spinning solution coming out of the spinneret passed through a 10 cm air gap and then entered the coagulation bath.

Following phase separation in a solidification bath, the newly formed hollow-fiber membrane was rinsed with pure water for 24 h at room temperature to eliminate any residual additives from the membrane filament surface. Subsequently, the hollow fibers were transferred to a 30% glycerin aqueous solution, where they were soaked for a period exceeding 24 h to prevent the collapse of membrane pores. Following the glycerin soak, the membrane filaments were air-dried at room temperature and stored in a zip-lock bag for future use.

### 2.3. Testing and Characterization

#### 2.3.1. Characterization of Membrane Morphology and Structure

A scanning electron microscope (SEM, S-4800; HITACHI, Tokyo, Japan) was utilized for the characterization of both the surface and cross-sectional morphologies of PES hollow-fiber membranes. The sample preparation process involved several steps: first, drying the membrane in a vacuum oven, followed by cutting a sample of suitable size and affixing it to conductive adhesive to prepare a surface sample. For cross-section samples, the membrane was rapidly quenched in liquid nitrogen, and the resulting cross-section sample was then mounted onto the conductive adhesive, with the cross-section slightly elevated above the surface of the sampling stage. Once sample preparation was completed, gold spraying was performed for 40 s to enhance the membrane’s conductivity, allowing the morphological characteristics of the sample to be observed through the instrument.

#### 2.3.2. Infrared Spectral Analysis

FT-IR is a commonly used method for qualitative and quantitative analysis of samples and for characterizing the chemical structure of polymers. The characteristics of FT-IR spectra can be used to determine the functional groups and chemical structure of polymer membranes. In this study, FT-IR spectroscopy (Nicolet 8700; Thermo Scientific, Waltham, MA, USA) was used to detect the incorporation of additives into polymer membranes. Transmission tests were performed on a membrane sample of appropriate size placed on a sample holder, using wavelengths ranging from 400 to 4000 cm^−1^.

#### 2.3.3. Contact Angle Test

The contact angle of pure water on the membrane surface was measured using a contact angle meter (OCA25; DataPhysics Instruments, Filderstadt, Germany), with the unit of measurement being degrees (°). The dried membrane was cut to an appropriate size and pasted onto a clean glass slide, which was then fixed onto the sample stage. A microsyringe was used to drop 3 μL of deionized water onto the sample surface at room temperature. To ensure the accuracy of the experimental results, we performed five measurements on different membranes prepared under the same conditions and averaged the final results.

#### 2.3.4. Mechanical Performance Tests

The mechanical properties of the membrane, encompassing tensile strength and elongation at break, were determined by a digital push–pull force tester (SH-20; Wenzhou Shandu Instrument Co., Ltd., Wenzhou, China). For the flat membrane sample, a membrane approximately 4 cm long and 0.5 cm wide was cut, and its thickness was measured with a thickness gauge (CLXL005; Syntek Electronic Technology Co., LTD, Wuxi, Jiangsu, China). The cross-sectional area of the membrane was then calculated. In the case of hollow-fiber membrane samples, the inner and outer diameters were measured under a stereomicroscope, and a length of approximately 4 cm of membrane filament was cut to calculate the cross-sectional area.

To conduct the tensile test, both ends of the membrane were fixed to the electronic push–pull force tester. The effective length was set to about 1 cm. The accurate effective length was recorded, and the sample was stretched at a speed of 30 mm/min until it broke. The tensile force displayed by the electronic tensile tester at breaking point and the length of the sample at that moment were recorded. The elongation at break and tensile strength of the sample can be calculated using Equations (1) and (2):

(1) $$\varepsilon =\frac{L-L_{0}}{L_{0}}\times 100\%$$

(2) $$\sigma =\frac{F}{S}$$

Of the variables, ε and σ are the elongation at break and mechanical strength of the sample in % and MPa, respectively; L_0_ and L are the initial accurate effective length of the sample and the length at tensile break in mm; F is the tensile force of the sample at the time of break in N; and S is the cross-sectional area of the sample in m^2^. In order to ensure the accuracy of the results of the experiments, the different membranes prepared under the same conditions were taken to be measured five times, and the average of the measured values was taken as the final result.

#### 2.3.5. Pure Water Flux Tests

The pure water flux of the membrane filament was measured by the internal pressure method with a staggered flow filtration device. Three hollow-fiber filaments of about 10 cm long were cut and installed into the membrane module; after pre-pressurization at 0.15 MPa for 10–15 min, the pure water flux was measured at 25 °C and 0.1 MPa, and the average value was taken after 10 min of testing time. The pure water flux was calculated by the following Equation (3):

(3) $$J=\frac{m}{1000\times \pi dl\cdot t}$$

where J represents the pure water flux at 0.1 MPa, unit: L/(m^2^·h); m is the mass of water through the membrane in time period t, unit: g; d is the inner diameter of the membrane filament, unit: m; l is the effective length of the membrane filament, unit: m; and t is the filtering time, unit: h.

#### 2.3.6. Molecular Weight Cutoff Test

A mixed solution containing dextran of different molecular weights (10,000 Da, 70,000 Da, 100,000 Da, 150,000 Da, and 500,000 Da, each with a concentration of 0.2 g/L and a total concentration of 1 g/L) was prepared. After mixing well, it was added into the feed tank, and the test was conducted by staggered flow filtration. The membrane area of the internal pressure hollow-fiber membrane module was about 56 cm^2^, and after 1 h of filtration at 0.1 MPa, the solutions on the permeate side and the retention side were started to be collected and analyzed by Shimadzu liquid chromatography (LC-20; SHIMADZU, Kyoto, Japan). Inlet conditions: pure water was used as the mobile phase at a flow rate of about 0.7 mL/min, the column temperature was set at 37 °C, an oscillometric detector was used, and a dextran standard sample was used for calibration. The molecular weight of dextran is considered as the MWCO of the membrane when the retention rate of dextran by the membrane is more than 90%.

#### 2.3.7. Zeta Potential Test

Usually, the charged state of the membrane surface is reflected by the potential on the surface of the sample membrane. In this experiment, an electrodynamic analyzer (SurPASS; Anton Paar, North Ryde, Australia) was used to measure the surface potential of the membrane. The membrane used for the test was a dry membrane. Before testing, the instrument was checked to see if the conductivity and pH meter needed to be calibrated, and the instrument was cleaned with high-purity water to a conductivity of less than 0.2 mS/m; the pH of the electrolyte solution was adjusted using 0.1 M HCl and NaOH to a range of 3–10, and then 0.015 g KCl was dissolved in 200 mL of deionized water to prepare a KCl electrolyte solution. The test device had to be cleaned with KCl solution before each test.

#### 2.3.8. Protein Static Adsorption Assays

A protein adsorption test was performed using neutral BSA or LYS as a test standard, which was stained by BCA or LZM protein kit and then measured by the absorbance method using an enzyme marker [33]. The experimental procedure is shown below:

1. A 0.5 g/L protein standard solution was prepared at pH 7. Four concentrations were then created through stepwise dilution: 0.1 g/L, 0.2 g/L, 0.3 g/L, and 0.4 g/L. The standard curve of the protein was plotted using the five concentrations.
2. To conduct the protein adsorption experiment, a pipette gun was used to add 2 mL of 0.5 g/L protein solution to a 24-well plate. Then, a PES hollow-fiber membrane with a membrane area of 0.5 cm^2^ was immersed into the protein solution. The membrane was incubated for 1 h at 37 °C in a shaker.
3. To determine protein adsorption, we used the BCA or LZM method. A BCA or LZM working solution was prepared by mixing reagent A and reagent B in a 50:1 ratio by volume.
4. To determine protein adsorption, 20 μL of each protein solution was taken from the 24-well plate and transferred to a 96-well plate. To determine protein adsorption, 20 μL of each protein solution was taken from the 24-well plate and transferred to a 96-well plate. To determine protein adsorption, 20 μL of each protein solution was taken from the 24-well plate and transferred to a 96-well plate. Some 200 μL of BCA or LZM working solution was added and the plate was incubated in a 37 °C shaker. The absorbance of the samples was measured using an enzyme labeling instrument (MultiskanTM FC; Thermo Fisher Scientific, USA) at a wavelength of 562 nm (BSA) or 281 nm (LYS). The protein concentration in the solution was calculated according to the standard working curve.

#### 2.3.9. Protein Ultrafiltration Experiments

A buffer solution was prepared by mixing NaCl, KCl, Na_2_HPO_4_·12H_2_O, and KH_2_PO_4_ in a mass ratio of 8.0:0.2:3.63:0.24, respectively. Distilled water was added to achieve a neutral pH, and the final volume was adjusted to 1 L. To the buffer solution, 0.5 g of either BSA or LYS was added without stirring and left to stand for 2 h. The resulting solution was then adjusted to a neutral pH by adding water.

**Figure 3:**
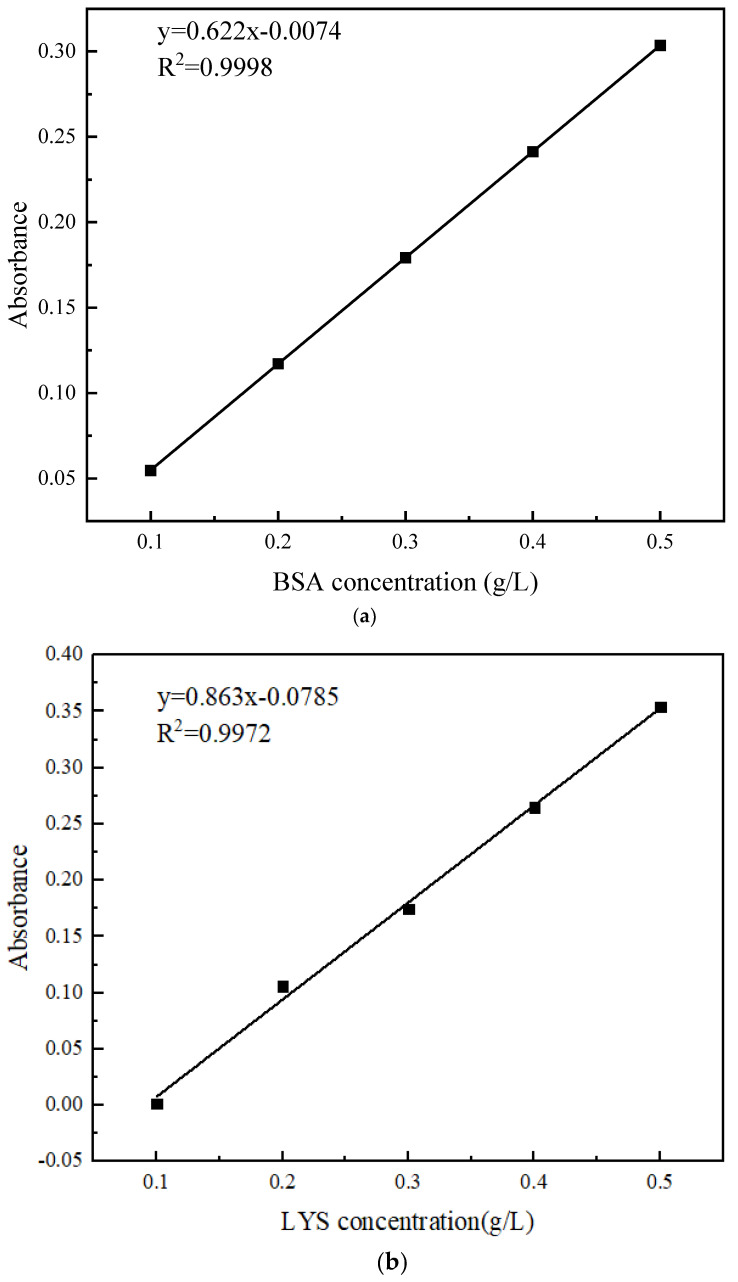
Standard curve: (**a**) BSA; (**b**) LYS.

Some 0.5 g/L of the protein buffer solution was diluted with pure water to obtain concentrations of 0.1, 0.2, 0.3, 0.4, and 0.5 g/L. The absorbance at 280 nm was measured using a UV-Vis spectrophotometer (UV-26001; SHIMADZU, Japan) and a standard curve of absorbance–protein concentration was plotted as depicted in Figure 3.

The method for determining protein retention was identical to that used for determining pure water flux. A 0.5 g/L protein solution was prepared and added to the feed tank. After filtration for a specific time at 25 °C and 0.1 MPa, the solution on the permeate and retention sides was collected and analyzed using a UV-Vis spectrophotometer to determine the absorbance at a wavelength of 280 nm. The protein concentration of the filtrate was determined by substituting the measured absorbance into the standard curve provided above. Subsequently, the retention rate of the PES ultrafiltration membrane on the protein solution was calculated using the following formula:

(4) $$R=\left(1-\frac{C_{1}}{C_{0}}\right)\times 100\%$$

where R is the membrane retention rate of protein in %, C_1_ is the concentration of permeate in g/L, and C_0_ is the concentration of feed solution in g/L.

Protein separation experiments were conducted using BSA and LYS. Ultrafiltration experiments were performed separately for BSA and LYS at pH 4 and 7, respectively. Additionally, a mixed solution of BSA and LYS (1:1) was also tested. The ultrafiltration experiments were carried out at 0.1 MPa and 25 °C with a protein concentration of 0.5 g/L. The protein concentration in the feed solution and permeate was measured using a UV-Vis spectrophotometer (λmax = 280 nm). The protein separation factor was calculated using Equation (5).

(5) $$\alpha_{\mathrm{LYS}/\mathrm{BSA}}=\frac{1-R_{\mathrm{LYS}}}{1-R_{\mathrm{BSA}}}$$

where R_LYS_ and R_BSA_ are the corresponding membrane retentions of protein (%).

## 3. Results and Discussion

### 3.1. Membrane Characterization

#### 3.1.1. Microstructure of Membranes

**Figure 4:**
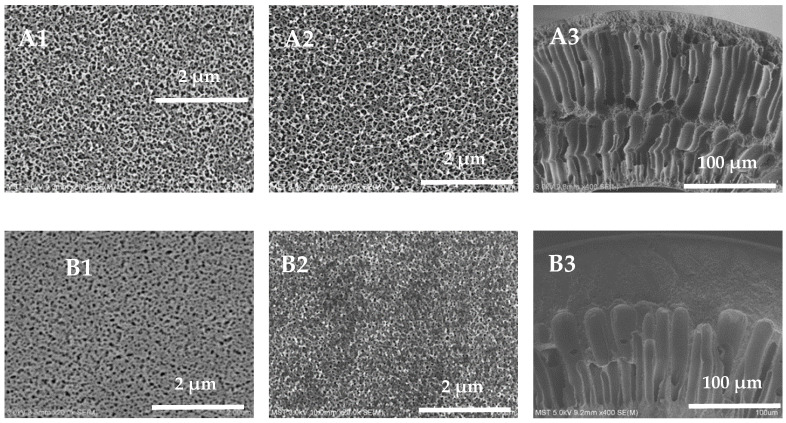

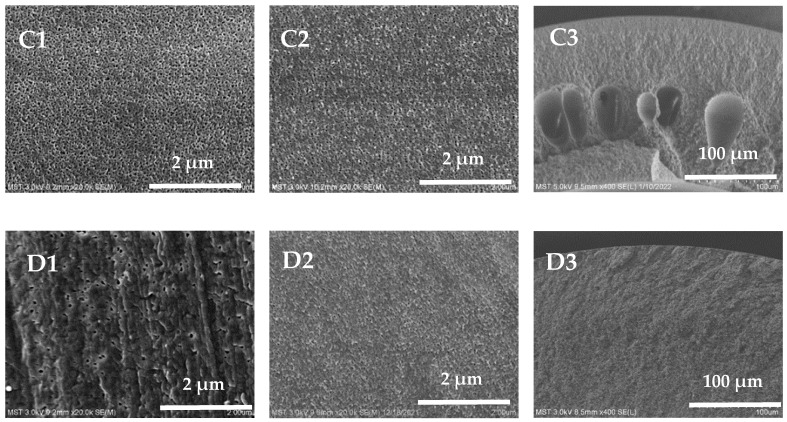
External surface, inner surface, and cross-section SEM images of the prepared membranes, (**A1**–**A3**) 5 wt%; (**B1**–**B3**) 12 wt%; (**C1**–**C3**) 16 wt%; (**D1**–**D3**) 20 wt%.

The external surface, inner surface, and cross-section of the hollow-fiber membranes are shown in Figure 4. All membrane samples exhibit an asymmetrical structure, with a dense surface separation layer supported by a porous sublayer. When the mass fraction of PVP-k30 is 5 wt%, the pore diameter on the external surface of the hollow-fiber membrane is larger and denser; when the mass fraction of PVP-k30 is 16 wt%, the pore diameter of the pores on the external surface of the membrane becomes smaller; and when the mass fraction of PVP-k30 is 20 wt%, only some of the holes with larger pore diameters can be seen on the external surface of the membrane. The overall trend is that with an increase in the content of PVP-k30, the pore diameter of the pores on the external surface of the hollow-fiber membrane gradually decreases. It is worth noting that when the PVP-k30 content in the spinning solution was 5%, the membrane exhibits a double-row finger-like pore structure in the cross-sectional view (Figure 4(A2)). PVP-k30 is a hydrophilic additive. When the PVP-k30 content in the spinning solution is low, the viscosity of the solution is low, and phase separation occurs rapidly after entering the coagulation bath, resulting in an increase in the number of finger-like pores. The PES/PVP/PEG hybrid system has greater affinity for water. This results in a prolonged exchange of solvent and non-solvent before gelation of the spinning solution, leading to a tendency for polymer-lean phase growth and agglomeration. Consequently, larger finger-like structures are formed [34].

As the concentration of PVP-k30 in the spinning solution increased from 12% to 20%, the number of finger-like pores in the cross-section of the PES hollow-fiber membrane decreased, and the proportion of sponge-like structure increased. At a PVP-k30 content of 20%, the cross-section SEM picture (Figure 4(D2)) shows a completely sponge-like structure. This is due to the increased viscosity of the spinning dope at higher concentrations, leading to slower phase separation and the presence of fewer finger-like pores in the cross-sectional structure [35].

#### 3.1.2. Chemical Composition of the Prepared PES Membranes

**Figure 5:**
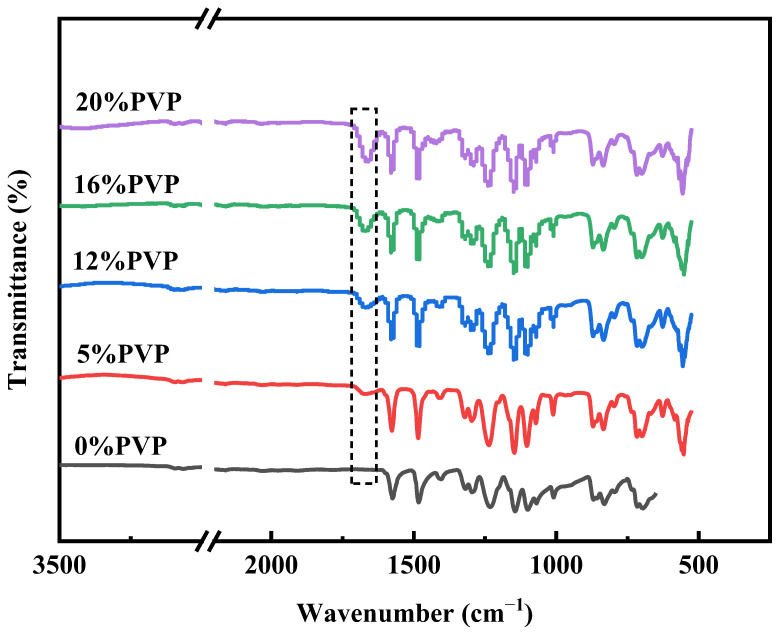
FT-IR spectra of PES membrane and PES/PVP/PEG hybrid membranes.

The chemical composition of the prepared membranes was analyzed by FTIR, and the spectra are shown in Figure 5. The FTIR spectra show absorption peaks at 1578 cm^−1^, 1485 cm^−1^, and 1240 cm^−1^, which are related to the benzene ring skeleton, carbon–carbon double-bond stretching, and aromatic ether groups, respectively. These unique peak positions are indicative of the PES membranes. The spectra also show that the PES/PVP/PEG hybrid membranes have a clear absorption peak at 1677 cm^−1^, which is determined to be the carbonyl group within the PVP-k30 molecule [36]. This shows that PVP was successfully doped into the PES membranes. Furthermore, the intensity of the peak increased with increasing PVP-k30 content.

#### 3.1.3. Pure Water Flux, BSA Retention, Mechanical Properties, and MWCOs

**Figure 6:**
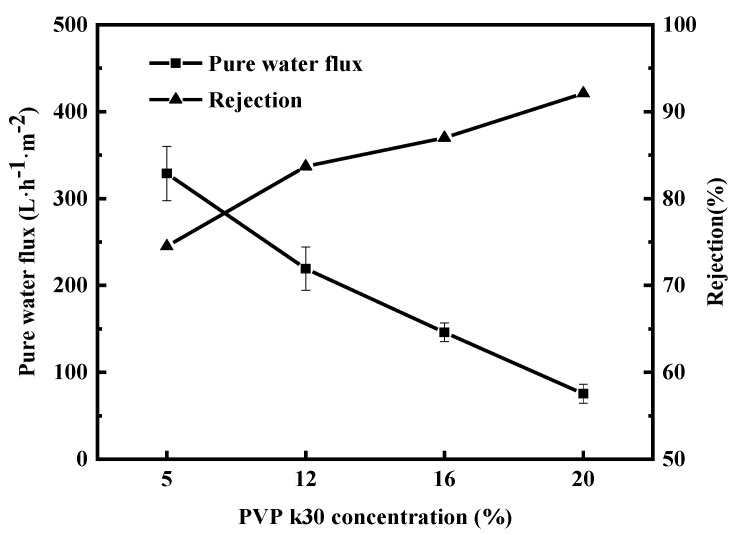
Effect of PVP-k30 content on pure water flux and BSA rejection of PES/PVP/PEG hybrid membranes.

Figure 6 shows the pure water flux and BSA retention rate of the membranes corresponding to four different PVP-k30 contents. The figure shows that pure water flux gradually decreased with increasing PVP-k30 content, while BSA retention rate shows an opposite trend. Notably, the pure water flux dropped sharply from 328 L·m^−2^·h^−1^ at 5% concentration to 234 L·m^−2^·h^−1^ at 12% concentration. When the concentration of PVP-k30 in the p solution was 20%, the flux decreased to 75 L·m^−2^·h^−1^. BSA retention increased from 74.5% at 5% PVP-k30 to 83.7% at 12% PVP-k30. When the PVP-k30 content in the spinning solution was 20%, the BSA retention rate further increased to 92.1%.

**Figure 7:**
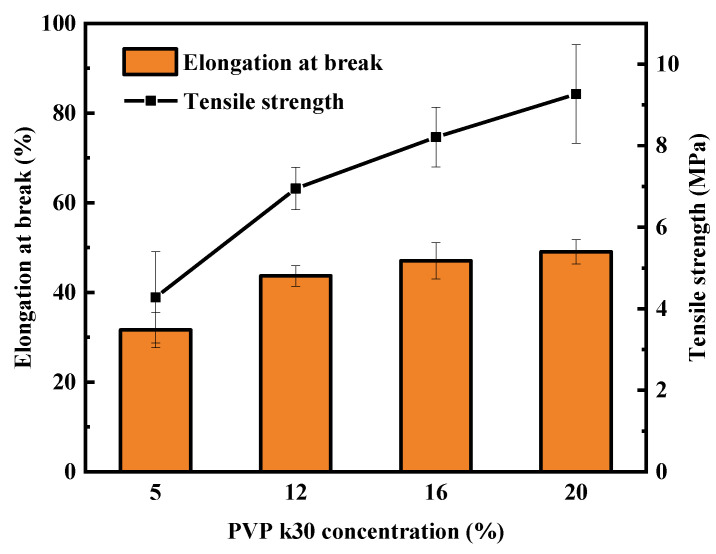
Effect of PVP-k30 content on mechanical properties of hollow-fiber membranes.

Figure 7 shows the effect of PVP-k30 content on the mechanical properties of hollow-fiber membranes. When the spinning solution contained 20 wt% PVP-k30, the PES/PVP/PEG membrane exhibited better mechanical properties. As the PVP-k30 content increased, both the tensile strength and the elongation at break increased; the tensile strength increased from 4.3 MPa to 9.3 MPa, while the elongation at break increased from 31.7% to 49.1%. Due to the higher concentration of PVP-k30 in the spinning solution, the macromolecular chains of the polymer became more entangled, leading to a reduction in the diffusion rate of the solvent and non-solvent. As a result, the sponge-like structure of the membrane cross-section became denser and the membrane porosity dropped, ultimately enhancing the mechanical strength of the PES/PVP/PEG membranes. Increasing the spinning solution concentration reduced non-solvent diffusion into the membrane within the coagulation bath. As a result, the formation of finger-like pores was reduced. When PVP-k30 with a concentration of 20 wt% was used, the membrane cross-section presented a homogeneous sponge-like structure, which significantly improved the mechanical strength of the hollow-fiber membrane.

**Figure 8:**
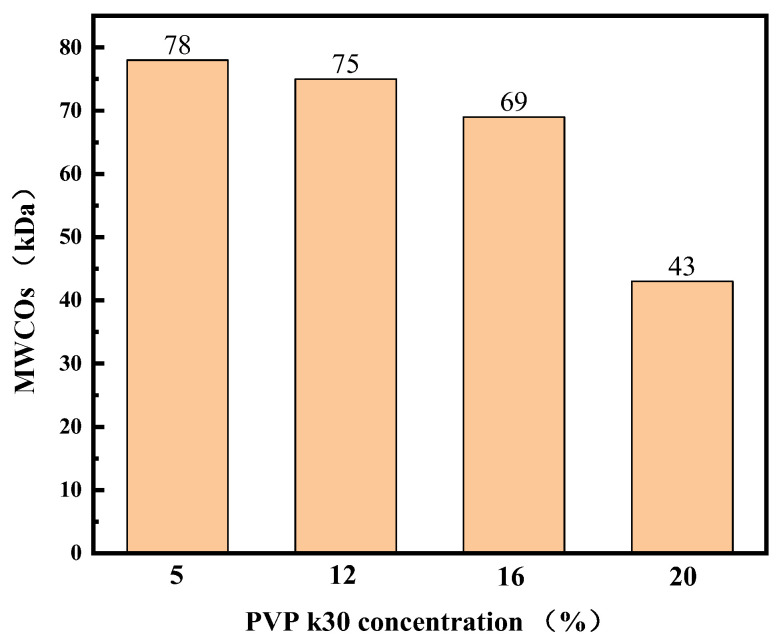
MWCOs of PES hollow-fiber membranes.

The MWCOs of the membranes were determined via ultrafiltration using a dextran mixture solution (Figure 8) and measured in the range of 43–78 kDa for the four hollow-fiber membranes. These values exceed the molecular weight of LYS. This result corresponds well with the surface morphology of these membranes and with the results of different phase separation processes. At the same time, the results of the molecular weight cutoff tests are consistent with the results of the pure water flux and BSA retention tests. This can be explained by the structural characteristics of the membranes.

#### 3.1.4. Contact Angle, Surface Charge, and Protein Adsorption

**Figure 9:**
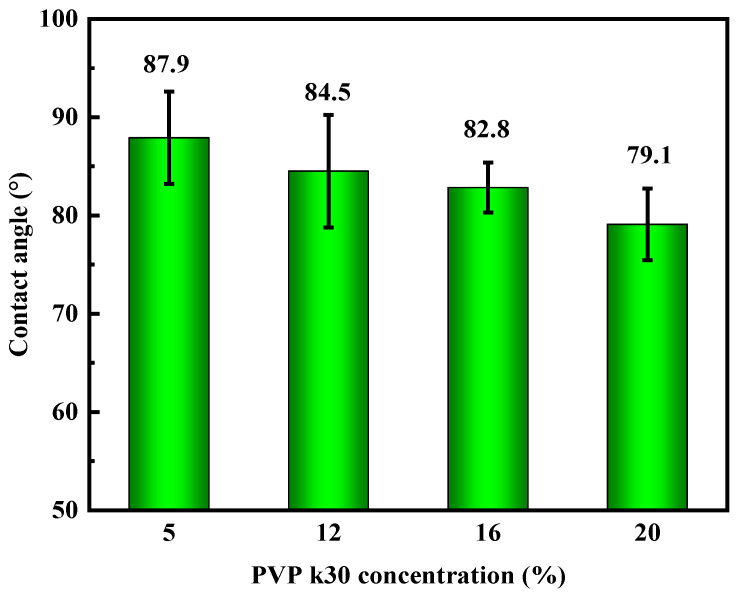
Effect of PVP-k30 content on contact angle of hollow-fiber membranes.

The impact of PVP-k30 content on the contact angle of the hollow-fiber membranes is presented in Figure 9. The data indicate that as the PVP-k30 content in the spinning solution increased, the contact angle of the hollow-fiber membranes decreased progressively, leading to an increase in hydrophilicity.

When the PVP-k30 content was 5%, the PES hollow-fiber membrane exhibited a contact angle of 87.9°, which decreased by 10% to 79.1° with a 20% PVP-k30 content. As a hydrophilic modifier, PVP contains a hydrophilic amide group in its molecular structure, and PVP-k30 enhances the hydrophilic properties of PES membrane to some extent [37]. Although the enhancement of hydrophilicity can increase the pure water flux and anti-fouling performance of the membrane, it ultimately improves its overall service life [38]. However, as mentioned above, as the content of PVP-k30 increases, the viscosity of the spinning solution increases, thereby delaying phase separation, causing the support layer structure of the membrane to become denser, which is not conducive to increasing the membrane flux. Improving the penetration of the pore structure of the membrane support layer may enable membranes with sponge-like pores to also have quite high pure water flux.

**Figure 10:**
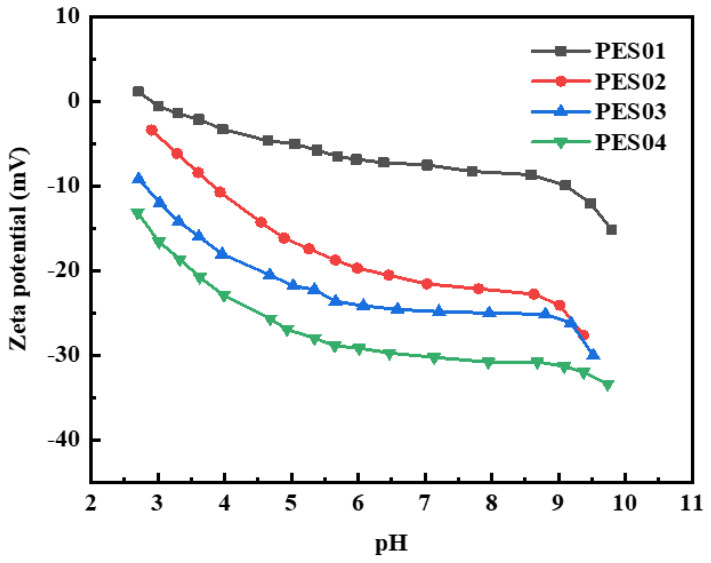
Zeta potential of the membranes at different pH values.

Since membrane charge plays a crucial role in selective separation, the zeta potential of the membrane surface was evaluated. Figure 10 shows the zeta potential values of hollow-fiber membranes at different pH levels. The isoelectric point (IEP) of the PES01 membrane is approximately pH 2.8. All four hollow-fiber membranes exhibited only negative zeta potential values in the pH range of 3–10. The absolute value of the membrane’s surface charge increased with increasing pH, but remained relatively stable at pH 7–9. The membrane surface was capable of absorbing varying amounts of anions (or cations) from the electrolyte solution. As a result, the membrane surface acquired a negative (or positive) net charge. The adsorption effect depends on the dielectric constant of the material. Negative charges are generally more prevalent on the membrane surface due to adsorption caused by stronger solvation of cations than anions. Cations prefer to remain in solution, whereas anions tend to solvate and therefore tend to accumulate on solid surfaces. At pH 7, the zeta potential values of PES01, PES02, PES03, and PES04 membranes were −7.5, −21.6, −24.9, and −30.2 mV, respectively. This indicates that an increase in the concentration of PVP k30 in the liquid solution causes protonation or deprotonation of functional groups on the membrane surface, and the zeta potential of the hollow-fiber membrane gradually becomes lower.

**Figure 11:**
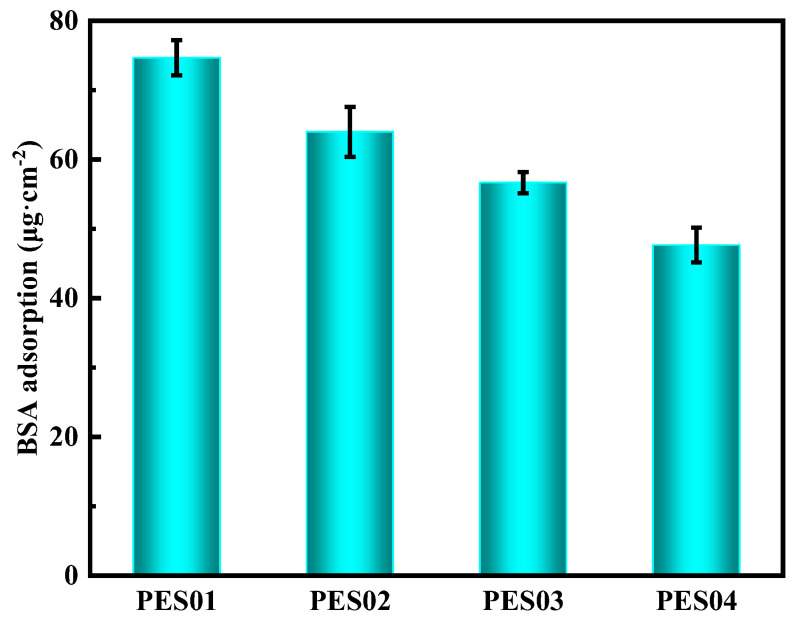
Protein adsorption of PES hollow-fiber membrane.

Figure 11 displays the fouling resistance of hollow fibre membranes, as determined through static adsorption experiments on BSA [39]. The protein concentration used was 0.5 g/L. The results show that under similar experimental settings, protein adsorption gradually decreased in the four hollow-fiber membranes. Of these, PES01 had the highest BSA adsorption capacity of 75 μg/cm^2^, while PES04 had the lowest BSA adsorption capacity of 48 μg/cm^2^. This phenomenon is consistent with changes in membrane contact angle—generally, protein adsorption decreases with increasing membrane hydrophilicity and, conversely, increases with decreasing hydrophilicity. The effect of membranes on protein adsorption was influenced by the hydrophilicity of the membranes and the electrostatic interactions between the protein and the membranes. At a pH of 7, BSA has a negative charge. The potential of PES01 was −7.5 mV, while that of PES04 was −30.2 mV. As the electrostatic repulsion between the negative charge state of BSA and the negatively charged membrane surface increased, BSA was more effectively blocked from the membrane surface.

### 3.2. Separation Properties of Bovine Serum Protein/lysozyme Binary Protein Solutions

Two protein models, BSA and LYS, were chosen for the study. The protein parameters can be found in Table 3. Ultrafiltration experiments for BSA and LYS were conducted at pH 4 and 7, respectively; a flow rate of 1.7 L/min; 0.1 MPa; and a temperature of 25 °C.

#### 3.2.1. Filtration Stability of Membranes in Monomer Protein Solutions

**Figure 12:**
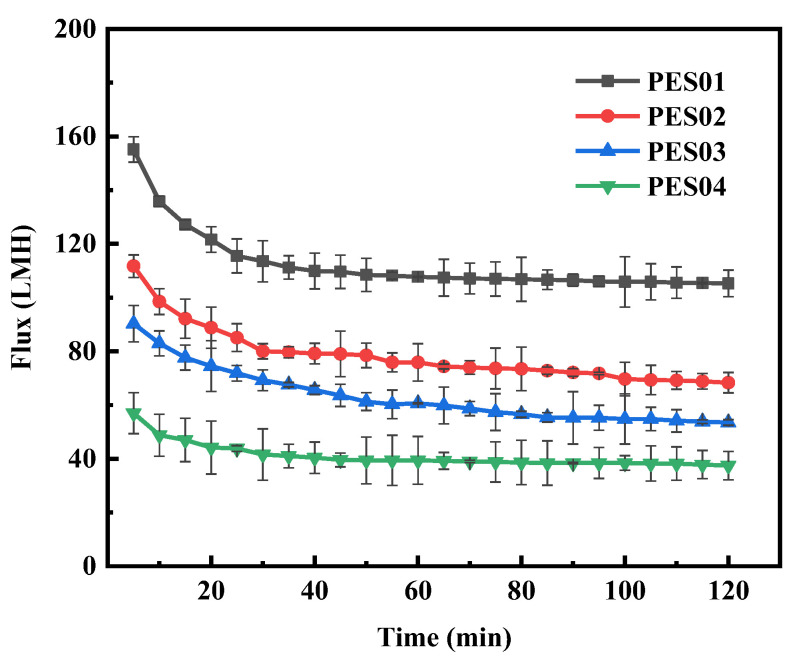
Flux variation of membranes in BSA solution.

**Figure 13:**
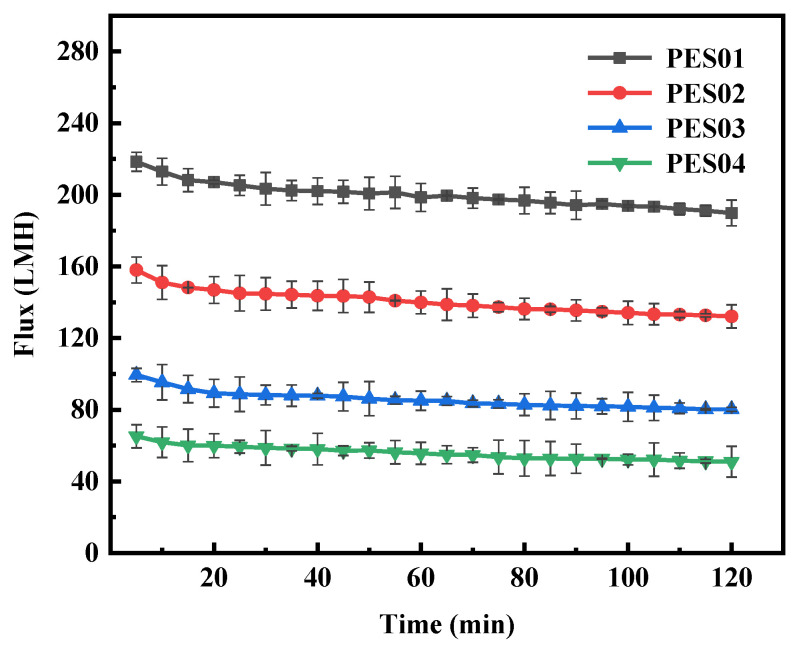
Flux variation of membranes in LYS solution.

Four hollow-fiber membranes were subjected to ultrafiltration in BSA and LYS solutions with a concentration of 0.5 g/L for 2 h, a pressure of 0.1 MPa, and a pH of 7. The changes in filtration flux over time for the four types of hollow-fiber membranes are shown in Figure 12 and Figure 13.

During BSA ultrafiltration, the membrane flux initially decreased rapidly and gradually stabilized over time. Notably, PES01 showed the most severe flux decrease, from an initial filtration rate of 155.1 LMH to 105.3 LMH, while PES04 had a smaller decrease, from an initial flux of 56.9 LMH to 37.5 LMH. In the first stage of ultrafiltration, smaller molecules in the feed solution passed through the membrane pores due to the pressure difference. Instead, larger molecules deposited on the membrane surface, causing a significant reduction in membrane flux. As ultrafiltration proceeded, it retained more large molecules. The adsorption of solutes on the membrane led to blockage of the pore channels, and the deposition of solutes thickened the gel layer on the membrane surface and increased fluid resistance. As a result, membrane fouling increased, leading to further reduction in filtration flux. However, the scouring effect of the fluid flow can cause concentration polarization, causing macromolecules to diffuse into the feed solution and detach from the membrane surface. When the detachment force is equal to the retention force on the membrane surface, the smooth operation of the ultrafiltration process is guaranteed. During ultrafiltration, LYS showed a larger initial flux and smaller flux decrease compared with BSA. Even after 20 min, the system maintained stable operation and the flux remained constant. This stability can be attributed to the smaller molecular size of LYS. Thanks to these factors, the membrane operated smoothly.

#### 3.2.2. Membrane Filtration in Mixed Solutions of Binary Proteins

**Figure 14:**
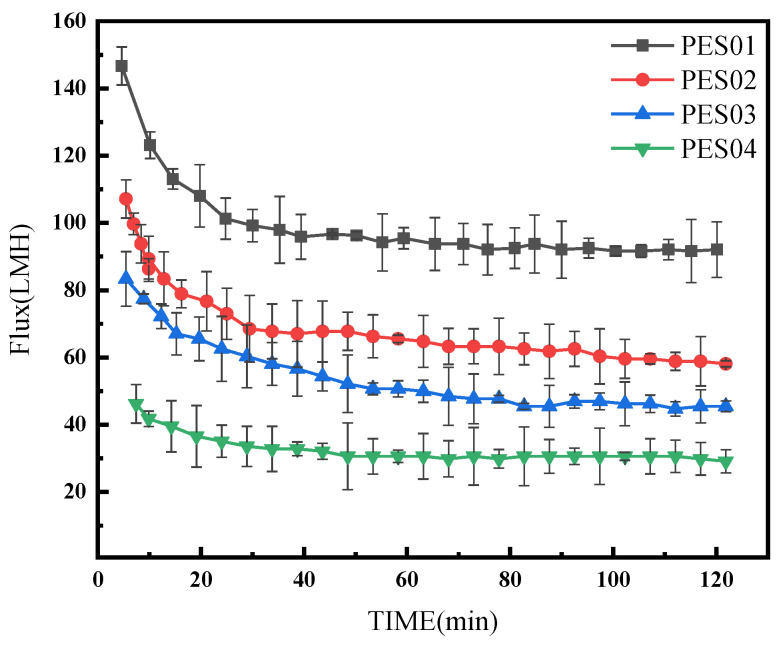
Membrane flux changes in a mixed solution of binary proteins.

The four hollow-fiber membranes were subjected to ultrafiltration using a mixture of BSA and LYS (1:1) with a total concentration of 1 g/L at a pressure of 0.1 MPa and pH 7 for 2 h. The change in filtration flux with time is shown in Figure 14.

In comparison with Figure 11 and Figure 12 under the same experimental conditions, the flux reduction caused by the BSA-LYS mixture was more severe than that caused by BSA or LYS alone. At pH 7, the negative charge of BSA and the positive charge of LYS created electrostatic attraction that accelerated protein attachment to the membrane. As a result, the binary mixed protein system suffered more severe fouling.

**Figure 15:**
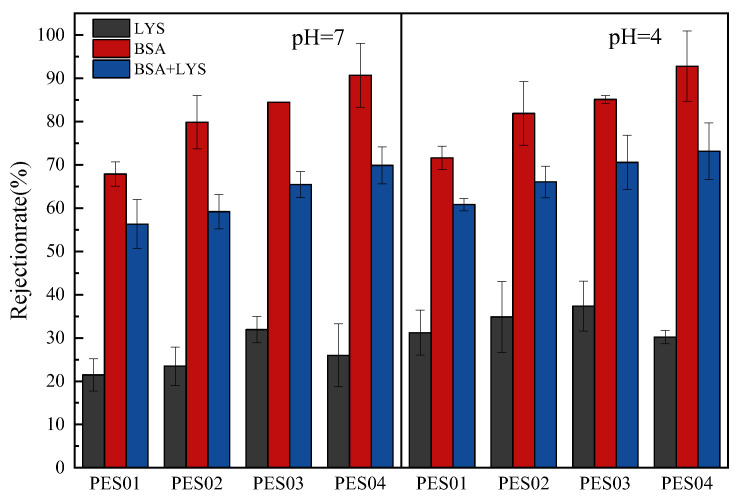
Retention rates of LYS, BSA, and LYS-BSA (1:1) mixed protein solutions.

The pH of the protein solution is a crucial factor in ultrafiltration separation employing charged membranes [9,40]. Acidic or basic groups on proteins and membranes modulate their charge, which can be changed by changing the pH of the solution. Ionization of acidic or basic groups creates repulsive or attractive interactions between the protein and the membrane surface. In this experiment, we determined the ultrafiltration separation of proteins at pH 4 and 7. The results are shown in Figure 15.

Due to its smaller size, LYS had lower retention than BSA on all four hollow-fiber membranes at both pH values. At pH 4 and 7, the LYS molecule (isoelectric point, IEP = 10) showed lower levels of retention. When positively charged, LYS molecules were attracted to the negatively charged surface of the PES membranes through electrostatic forces. However, there was also electrostatic repulsion between LYS molecules attached to the membrane surface and LYS molecules in solution. When the pH of the feed solution was 4, the negative charge on the membrane surface was reduced, resulting in a reduction in the electrostatic attraction with LYS and an increase in the retention rate. At a pH of 4, BSA molecules were positively charged. This resulted in an electrostatic attraction between the negatively charged membrane surface, causing the membrane’s pore size to decrease. Therefore, this increased retention rates. It can be seen from the figure that compared to other hollow-fiber membranes, PES04 has the highest retention rate for BSA, while the retention rate for LYS is relatively low. When pH = 7, the retention rate of PES04 on BSA is 91%, and the retention rate of LYS is 18%. Furthermore, the separation factors of PES01, PES02, PES03, and PES04 were 2.48, 3.86, 4.41, and 9.11, respectively. When the pH was set to 4, PES04 retained 93% of BSA and only 23% of LYS. In addition, the separation factors of PES01, PES02, PES03, and PES04 were 2.45, 3.79, 4.31, and 11, respectively. The experimental results indicate that PES04 demonstrated superior performance in separating LYS and BSA. It is worth noting that when the same membrane was used to filter protein solutions of varying pH values, the separation factors exhibited significant differences. This observation suggests that adjusting the pH of protein solutions to match the isoelectric points of different proteins can effectively optimize purification. Therefore, in practical applications, regulating the pH of the solution according to the differences in protein isoelectric points would be an effective strategy to improve the efficiency and effectiveness of protein purification [41].

## 4. Conclusions

PES was used as a membrane material, and PVP-k30 and PEG 400 were used as water-soluble additives. Using DMAc as a solvent, PES hollow-fiber membranes were prepared by the non-solvent diffusion phase separation method. This paper systematically studied the impact of membrane production conditions on the structure, morphology, and performance of PES hollow-fiber membranes. Two types of proteins have been used in protein separation studies—BSA and LYS. Four PES hollow-fiber membranes with different support layers were tested, and the results provided the following insights:

PES hollow-fiber membranes were prepared using the non-solvent diffusion phase separation method. When the PVP-k30 content was increased to 20%, the cross-sectional structure of the PES hollow-fiber membrane became sponge-like, the mechanical strength increased to 10.16 MPa, the contact angle decreased to 79.1°, and the hydrophilicity was enhanced.

Two protein models with different molecular weights and sizes, namely BSA and LYS, were selected as research objects. We studied the protein separation performance of PES hollow-fiber membranes by conducting molecular weight cutoff, zeta potential, and protein static adsorption tests on the membrane. Studies have found that the effect of a membrane on protein adsorption depends not only on its hydrophilicity but also on the electrostatic interaction between the protein and the membrane. The optimization of protein purification can be achieved by adjusting the pH of the solution to account for the difference in isoelectric points of various proteins [42,43,44]. This approach enhances the efficiency and selectivity of purification, resulting in higher purity of the target proteins. During the ultrafiltration process, concentration polarization, membrane pore clogging, and gel layer formation would lead to a reduction in filtration flux. This study found that the PES hollow-fiber membrane with a full sponge-like structure exhibited excellent anti-fouling ability and could effectively separate BSA and LYS.

Given the wide application of membrane technology, improving the PES membrane structure of various protein separation systems is of great scientific and practical significance. This includes improving separation accuracy, reducing membrane fouling in different separation systems, developing effective cleaning methods, and extending the service life of PES membranes to enhance anti-fouling performance.

## Figures and Tables

**Table 1:** Manufacturing parameters for membranes.

Membranes	Dope Compositions (wt%)			
	PES	PVP-k30	PEG	DMAc
PES00	20	0	8	72
PES01	20	5	8	67
PES02	20	12	8	60
PES03	20	16	8	56
PES04	20	20	8	52

**Table 2:** Hollow-fiber membrane preparation process conditions.

Process Conditions	Parameters
Feed tank operating temperature (°C)	60
Bore liquid tank operating temperature (°C)	25
Gear pump operating temperature (°C)	60
Spinneret operating temperature (°C)	60
Bore liquid composition	H_2_O
Spinning solution pump speed	4 m/s
Bore liquid pump speed	10 m/s
Coagulation bath	H_2_O
Temperature of the coagulation bath	20 °C
Air gap	10 cm

**Table 3:** Protein model parameters.

Parameters	LYS	BSA
Molecular weight (Da)	14,300	68,000
Molecular size (nm)	4.5 × 3 × 3	14 × 3.8 × 3.8
Isoelectric point	10.8	4.7

## Data Availability

Data are contained within the article.
